# Trauma exposure, post-traumatic stress disorder and alcohol and other drug use among Australian public safety personnel

**DOI:** 10.1177/00048674251324814

**Published:** 2025-03-15

**Authors:** Jayden Sercombe, Amelia Henry, Coleen Leung, Matthew Sunderland, Christina Marel, Emma Barrett, Ashleigh K Morse, Mina Askovic, Alana Fisher, Mary-Lou Chatterton, Logan Harvey, Natalie Peach, Maree Teesson, Katherine L Mills

**Affiliations:** 1 The Matilda Centre for Research in Mental Health and Substance Use, The University of Sydney, Sydney, NSW, Australia; 2 The eCentreClinic, School of Psychological Sciences, Macquarie University, Sydney, NSW, Australia; 3 School of Public Health and Preventive Medicine, Monash University, Melbourne, VIC, Australia

**Keywords:** Public safety personnel, alcohol and other drug use, trauma, post-traumatic stress disorder, workplace mental health

## Abstract

**Objective:**

This study investigated the rates of workplace trauma exposure, probable post-traumatic stress disorder (PTSD) and alcohol and other drug use among Australian public safety personnel (PSP). It also identified factors associated with hazardous or harmful alcohol and other drug use.

**Method:**

Data were collected through an online survey distributed to PSP in three Australian agencies between May and October 2021. A total of 539 PSP completed the survey. The survey included questions about demographic and work-based characteristics, trauma exposure, PTSD and alcohol and other drug use.

**Results:**

Most participants (86.8%) had experienced at least one type of traumatic event at work. Probable PTSD was identified in 39.4% of respondents. Hazardous or harmful alcohol use was reported by 33.1% of participants, while 13.3% reported harmful drug use. Analyses revealed that probable PTSD and higher numbers of workplace traumatic event types were significantly associated with greater odds of hazardous or harmful alcohol use (ORs 1.88 and 1.04, respectively). Identifying as female and meeting criteria for probable PTSD was associated with greater odds of harmful drug use (OR = 1.86) and identifying as male with lower odds of harmful drug use (OR = 0.23).

**Conclusion:**

The study highlights the high prevalence of trauma exposure, probable PTSD and hazardous or harmful substance use among Australian PSP. The findings suggest a need for targeted interventions to address the mental health and substance use challenges in this population, particularly those aimed at mitigating the effects of workplace trauma and providing support for PTSD and substance use disorders.

## Introduction

Mental illness is the leading cause of workplace absenteeism and prolonged disability in most developed countries (Murray et al., 2012). Each year, depression and anxiety are estimated to cost the world economy over US$1 trillion, driven by workplace absence (Chisholm et al., 2016). For some employees, the nature of the work itself may increase the risk of mental illness, such as those who experience high rates of trauma exposure (Harvey et al., 2017).

Public safety personnel (PSP), who provide critical services related to ensuring the wellbeing and safety of individuals and communities, are often exposed to trauma and high-stress situations in the workplace (Carleton et al., 2019; Kyron et al., 2019b). In Australia, there are over 120,000 PSP including paramedics, police, firefighters, correctional officers and dispatchers (Edgelow et al., 2023; Kyron et al., 2019a). The unique occupational challenges experienced by PSP put them at increased risk of developing mental health and substance use concerns (Patel et al., 2023). Traumatic events can happen directly to the worker (e.g. physical assault, fighting a bushfire) or may be observed by them (e.g. witnessing severe human suffering or death). Repeated exposure to traumatic content as part of workplace duties is particularly relevant for PSP and is recognised as a qualifying antecedent of PTSD (American Psychiatric Association, 2022; Pai et al., 2017). The prevalence of PTSD in PSP is 10%, more than double that of the general population (Berger et al., 2012; Koenen et al., 2017). This rate of PTSD is concerning as the disorder can potentially lead to subsequent problematic alcohol and other drug use.

PTSD and traumatic event exposure have been closely linked with problematic alcohol and other drug use in PSP, particularly in research conducted in North America (Bonumwezi et al., 2022; Patel et al., 2023). For example, Carleton et al. (2019) estimated that over 90% of Canadian PSP had been exposed to trauma through their work, and those who had been exposed to sudden violent death through their role had 3.4 times the likelihood of screening positive for an alcohol use disorder, compared to those with no exposure. One possible explanation for this trend is that the consumption of alcohol, and to some extent other drugs, is often perceived as a culturally appropriate method of coping with stress among PSP exposed to trauma (Gulliver et al., 2019; Useche et al., 2019). However, the use of alcohol and other drugs to manage traumatic stress can be problematic and potentially lead to the development of substance use disorders. This method of coping can be further compounded by the role that substance use disorders have in exacerbating symptoms of PTSD (Turner et al., 2018), creating a cycle that is difficult to break.

It is well known that these workers have high rates of traumatic event exposure but the relationship of these factors with alcohol and other drug use is understudied. There is very little research outside of North America. In Australia, a large survey of the mental health of Australian PSP conducted by Beyond Blue (*n* = 14,868) revealed that of those with probable PTSD, 25% were engaging in binge drinking and 5% had used illicit drugs in the past month (Kyron et al., 2019a). However, there remains a critical need for more research into alcohol and other drug use in this population and factors associated with problematic use (Varker et al., 2018). A greater understanding is imperative to informing interventions and structures needed to support and sustain this essential workforce.

This elevated prevalence underscores the need for effective interventions and support systems for PSP, especially considering the potential for subsequent issues such as problematic alcohol and other drug use. To address this gap, this study aimed to assess rates of trauma exposure, probable PTSD and alcohol and other drug use in a sample of Australian PSP, and to investigate correlates of hazardous or harmful alcohol and other drug use including demographic characteristics, work-based factors, workplace trauma exposure and PTSD. Identifying key factors linked to substance use can inform the development of targeted support and prevention strategies.

## Methods

### Procedure and participants

Data were collected through an online survey in Qualtrics, distributed between May and October 2021. To be eligible for the study, participants needed to (1) be at least 18 years old and (2) have been employed by one of three Australian public safety agencies either currently or within the previous year. Recruitment was conducted by disseminating study advertisements through internal agency channels including staff portal banners and all-staff emails and through targeted social media advertisements.

The survey was pilot tested by representatives from each agency, who provided recommendations and assisted with the development and implementation of recruitment activities. As confidentiality was identified as a concern, it was stressed to PSP that the researchers were independent from their workplace and that respondents would remain anonymous. All participants in this study provided informed written consent prior to their involvement. Ethical approval was obtained from the University of Sydney Human Research Ethics Committee (protocol number: 2022/891) and the Ethics Review Committee (RPAH Zone; 2021/ETH00166) of the Sydney Local Health District.

### Measures

Participants responded to questions regarding their demographic and work-based characteristics; trauma exposure; PTSD; and alcohol and other drug use.

#### Demographic and work-based characteristics

The questionnaire asked about demographic information, including age and gender. Participants were also asked about their work-based characteristics, including their role, rurality and length of service. Role was categorised as frontline (e.g. on-the ground responders, dispatchers, or call-takers) or non-frontline (e.g. administration or management roles). The geographical location of work was assessed as per the Australian Bureau of Statistics (ABS, 2022) definitions. For full information on response options and results, see Table 1.

**Table 1.** Characteristics of the sample.

Personnel characteristics		*n* (%)
Age (years)	<35	194 (36.0)
	35–44	105 (19.5)
	45–54	127 (23.6)
	⩾55	113 (21.0)
Gender	Male	265 (49.4)
	Female	268 (50.0)
	Non-binary^a^	3 (0.6)
Role^b^	Frontline	351 (65.1)
	Non-frontline	188 (34.9)
Geographic region of work^c^	Major urban area	312 (58.2)
	Other urban or country area	177 (33)
	Small country or rural/remote area	47 (8.7)
Length of service	<3 years	110 (20.4)
	3–5 years	82 (15.2)
	6–10 years	89 (16.5)
	*>*10 years	258 (47.9)
Probable PTSD (PC-PTSD-5)	Below cutoff	320 (60.6)
	Probable PTSD	208 (39.4)
Alcohol use: AUDIT	Abstinent	56 (10.4)
	Low-risk	304 (56.4)
	Hazardous	121 (22.5)
	Harmful	25 (4.6)
	Dependent	33 (6.1)
Drug use: DUDIT	Abstinent	433 (80.3)
	Low-risk	30 (5.6)
	Harmful	68 (12.6)
	Dependent	4 (0.7)

SD = Standard Deviation. NA = Not Applicable. PTEs = Potentially Traumatic Events. AUDIT = Alcohol Use Disorders Identification Test. DUDIT = Drug Use Disorders Identification Test. Primary Care PTSD Screen for *DSM*-5 = PC-PTSD-5.

a Non-binary/different identity participants were not included in analyses due to low cell counts.

b Frontline (e.g. on-the ground responders, dispatchers, or call-takers) or non-frontline (e.g. administration or management roles)

c Major urban area (population of 100,000–1 million), other urban or country area (population of 1,000–99,999), small country or rural/remote area (population of less than 1,000).

#### Traumatic events

The Life Events Checklist for *DSM*-5 (LEC-5; Weathers et al., 2013) is a self-report scale designed to measure trauma history. The LEC-5 assesses the occurrence and context of exposure to 17 traumatic events (e.g. physical assault, sudden accidental death). The LEC has been widely used in PSP research (e.g. Carleton et al., 2019) and is a reliable and valid measure of trauma exposure (Weis et al., 2022). Based on feedback from participating organisations advising of common experiences of employees, two additional items were incorporated for a total of 19 events: ‘*threats of violence*’ and ‘*stalking*’. Regarding whether they had been exposed to each event, participants selected one or more of the following: ‘*happened to me*’, ‘*witnessed it*’, ‘*part of my job*’. In this study, the focus was on traumatic events experienced through work. As such, the LEC-5 was scored by summing up all events participant’s endorsed as having occurred as part of their job, based on the recommendations of Weis et al. (2022).

#### Probable PTSD

The Primary Care PTSD Screen for *DSM*-5 (PC-PTSD-5; Prins et al., 2016) was used to identify probable PTSD. The instrument is a 5-item self-report measure and assesses PTSD symptoms, such as avoidance, hyperarousal and numbing, occurring in the past month. Respondents answer ‘*yes*’ or ‘*no*’ to each item for a total score range of 0-5, with scores of over ⩾ 3 indicating probable PTSD. The measure has demonstrated strong psychometric properties in populations with high trauma exposure (Bovin et al., 2021) and had good reliability in this study (α = 0.79).

#### Alcohol use

The World Health Organisation’s (WHO) Alcohol Use Disorders Identification Test (AUDIT; Saunders et al., 1993) was used to measure alcohol-related harm. The questionnaire consists of 10 items about alcohol consumption and related problems, with responses scored from 0-4. Total scores range from 0 to 40, with higher scores indicating greater risk for alcohol-related problems. The AUDIT is a widely utilised instrument and has high reliability and validity (Babor et al., 2001). As per the WHO’s guideline manual (Babor et al., 2001), participants were categorised as abstinent (no alcohol use), engaging in low-risk use (scores: 1-7), hazardous use (8-15), harmful use (16-19), or dependent use (⩾ 20). In this study, the AUDIT demonstrated good reliability (α = 0.85).

#### Drug use

The Drug Use Disorders Identification Test (DUDIT; Berman et al., 2005) is an 11-item self-report scale that assesses drug use, drug-related problems and drug dependence over the past 12 months. Similar to the AUDIT, item responses are scored from 0-4. Total scores range from 0-44, with higher scores indicating greater risk for drug-related problems. Participants were categorised as either abstinent (no drug use), engaging in low-risk drug use (scores of 1 for women, 1-5 for men), harmful drug use (scores of 2-24 for women or 6-24 for men), or dependent drug use (⩾ 25). The DUDIT has demonstrated good reliability and validity in previous research (Berman et al., 2005) and showed excellent reliability in the current study (α = .90).

### Data analysis

The data were cleaned and analysed using R version 4.2.1 in Rstudio (R Core Team, 2023). Descriptive statistics were examined for demographic characteristics, work-based factors, traumatic event exposure, probable PTSD and substance use variables. For continuous variables, means and standard deviations were calculated, while categorical variables were characterised by participant counts and proportions within each category. Scores on the AUDIT and DUDIT were transformed into binary variables based on hazardous and harmful use (Yes/No). For the AUDIT, scores of ⩾ 8 are indicative of hazardous and harmful alcohol use. For the DUDIT, females scoring ⩾ 2 and males scoring ⩾ 6 indicate harmful drug use (Berman et al., 2005). A series of univariate binomial logistic regressions were completed to investigate the relationships between each variable and the binary outcomes of AUDIT risk level and DUDIT risk level. Two multivariable binomial logistic regressions (one with AUDIT risk level as the outcome and one with DUDIT risk level as the outcome) were undertaken with all independent variables included as covariates to account for confounding effects. The significance level for analyses was set at *p* < .05.

## Results

### Sample characteristics

In total, 539 PSP completed the survey. Around half of participants were male (49.7%) and 45 or older (44.6%). Slightly under half of participants (47.9%) had been working at their relevant agency for 10 years or more. The majority of participants reported being frontline workers (65.1%), for example paramedics, firefighters, call-takers or dispatchers. More than half of participants (58.2%) reported working in a major urban area. Full demographics and role characteristics can be found in Table 1.

## Rates of trauma exposure and probable PTSD

### Exposure to traumatic events

**Figure 1.**
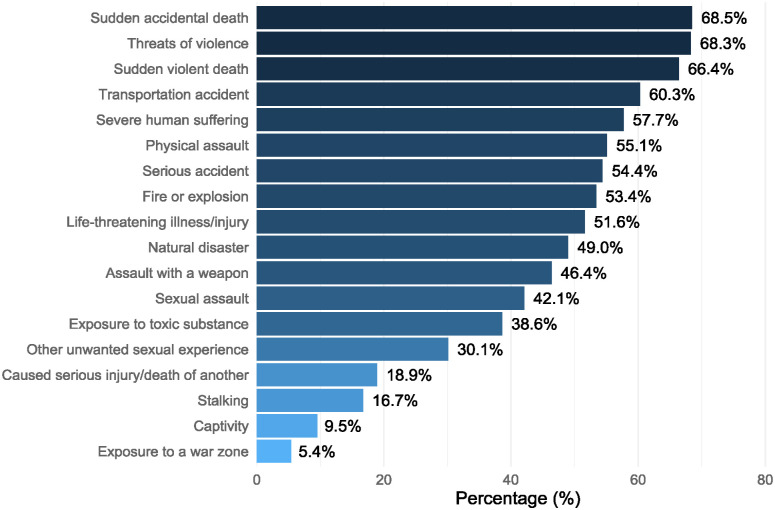
Proportion of the sample that reported experiencing each type of traumatic event type through work.

In the sample, 86.8% had experienced at least one type of traumatic event as part of their job. The mean number of types of events experienced in the workplace was 8.4 (SD = 5.5), most commonly having exposure to sudden accidental death (68.5%); threats of violence (68.3%) and sudden violent death (66.4%; Figure 1).

### Probable PTSD

The mean score on the PC-PTSD-5 was 2.1 (*SD* = 1.8). Overall, 39.4% of participants were identified as having probable PTSD.

## Rates of alcohol and other drug use

Table 1 shows the number and proportion of participants in each AUDIT and DUDIT risk category. The mean overall AUDIT score was 7.0 (SD = 6.6). While two thirds (66.6%) reported being abstinent or drinking at low-risk levels, 33.1% fell into the category of hazardous or harmful patterns of alcohol use.

One in five (19.7%) participants reported using other drugs in the past 12 months. The most commonly used drugs were cocaine (8.2%), followed by cannabis (7.8%) and benzodiazepines (5.8%). The average DUDIT score for the total sample was 1.3 (*SD =* 4.1); 13.3% were classified as using at levels indicative of harmful or dependent drug use.

## Factors associated with hazardous and harmful alcohol and drug use

### Hazardous and harmful alcohol use

No demographic or work-related variables (including gender, age, rurality, role, or length of service) exhibited significant independent associations with alcohol risk category in the univariable regression analyses. However, the odds of hazardous and harmful use were positively associated with probable PTSD and the number of workplace traumatic event types experienced (see Table 2).

**Table 2.** Logistic regressions showing associations between PSP characteristics and hazardous and harmful alcohol use.

	Univariate analyses		Multivariate analysis	
	OR (95% CI)	p-value	OR (95% CI)	p-value
**Gender**				
*Female*	—		—	
*Male*	1.43 (1.00 to 2.06)	.053	1.27 (0.85 to 1.89)	.245
**Rurality**		.339		.580
*Major urban area*	—		—	
*Other urban or country area*	1.34 (0.91 to 1.97)	.142	1.25 (0.82 to 1.90)	.296
*Rural/remote/small country*	1.16 (0.59 to 2.20)	.652	1.09 (0.54 to 2.15)	.798
**Role**				
*Frontline*	—		—	
*Non-frontline*	1.02 (0.70 to 1.48)	.914	1.16 (0.75 to 1.78)	.499
**Length of service**		.099		.638
*< 3 years*	—		—	
*3–5 years*	1.02 (0.53 to 1.95)	.942	1.08 (0.55 to 2.11)	.825
*6–10 years*	1.49 (0.81 to 2.75)	.197	1.27 (0.65 to 2.48)	.477
*>10 years*	1.68 (1.04 to 2.79)	.039	1.56 (0.78 to 3.16)	.214
**Age**		.375		.377
*<35*	—		—	
*35–44*	1.49 (0.91 to 2.44)	.115	1.03 (0.56 to 1.86)	.926
*45–54*	1.14 (0.71 to 1.84)	.582	0.70 (0.36 to 1.34)	.289
*55+*	0.96 (0.58 to 1.59)	.878	0.62 (0.29 to 1.28)	.198
**Workplace traumatic events**	1.06 (1.03 to 1.10)	**<.001**	1.04 (1.00 to 1.08)	**.039**
**Probable PTSD**				
*No*	—		—	
*Yes*	1.90 (1.32 to 2.75)	**<.001**	1.88 (1.29 to 2.76)	**.001**

OR = Odds Ratio, CI = Confidence Interval, PTSD = Post-Traumatic Stress Disorder.

In the multivariable model (see Table 2), these associations remained significantly associated with hazardous and harmful alcohol use. Workplace traumatic events displayed a significant relationship, where each additional experienced event was associated with higher odds of harmful alcohol use (OR = 1.04, 95% CI [1.00 to 1.08], *p* = .039). In addition, participants with probable PTSD had higher odds of hazardous and harmful alcohol use compared to those without probable PTSD (OR = 1.88, 95% CI [1.29 to 2.76], *p* = .001).

### Factors associated with harmful drug use

In the univariable analyses predicting drug use risk category (see Table 3), males were significantly less likely to report harmful drug use compared to females. Length of service was also significantly related to harmful drug use, where workers with more than 10 years of service had lower odds of harmful drug use, when compared to those with less than 3-5 years. In addition, age was significantly associated with harmful drug use, where participants aged 45-54 and 55 years and older had decreased odds of harmful drug use, compared to those aged younger than 35. Finally, probable PTSD was associated with harmful drug use. No other significant associations were found.

**Table 3.** Logistic regressions showing associations between PSP characteristics with harmful drug use.

	Univariate analyses		Multivariate analysis	
	OR (95% CI)	p-value	OR (95% CI)	p-value
**Gender**				
*Female*	—		—	
*Male*	0.18 (0.09 to 0.33)	**<.001**	0.23 (0.11 to 0.43)	**<.001**
**Rurality**		.779		.712
*Major urban area*	—		—	
*Other urban or country area*	0.83 (0.47 to 1.44)	.520	0.95 (0.51 to 1.74)	.873
*Rural/remote/small country*	1.06 (0.41 to 2.39)	.892	0.68 (0.25 to 1.66)	.421
**Role**				
*Frontline*	—		—	
*Non-frontline*	0.86 (0.50 to 1.45)	.587	1.06 (0.55 to 1.98)	.867
**Length of service**		**<.001**		.331
*<3 years*	—		—	
*3–5 years*	0.62 (0.29 to 1.27)	.201	0.68 (0.30 to 1.47)	.330
*6–10 years*	0.66 (0.32 to 1.33)	.257	0.84 (0.38 to 1.83)	.662
*More than 10 years*	0.24 (0.13 to 0.47)	**<.001**	0.46 (0.18 to 1.12)	.087
**Age**		**<.001**		.372
*<35*	—		—	
*35–44*	0.68 (0.35 to 1.27)	.240	0.97 (0.45 to 2.05)	.947
*45–54*	0.26 (0.11 to 0.55)	**.001**	0.46 (0.16 to 1.17)	.114
*55+*	0.29 (0.12 to 0.61)	**.002**	0.67 (0.22 to 1.94)	.473
**Workplace traumatic events**	0.98 (0.94 to 1.03)	.424	1.01 (0.95 to 1.07)	.812
**Probable PTSD**				**.024**
*No*	—		—	
*Yes*	1.73 (1.04 to 2.86)	**.033**	1.86 (1.09 to 3.20)	

OR = Odds Ratio, CI = Confidence Interval, PTSD = Post-Traumatic Stress Disorder.

After including all variables in the fully adjusted logistic regression model, only gender and probable PTSD remained significantly associated with drug use risk category. In terms of gender, males had lower odds of harmful drug use compared to females (OR = 0.23, 95% CI [0.11 to 0.43], *p* < .001). Further, those identified as having probable PTSD had higher odds of harmful drug use (OR = 1.86, 95% CI [1.09 to 3.20], *p* = .024). Factors such as rurality, role, length of service, age and workplace traumatic events did not exhibit significant associations with harmful drug use (see Table 3).

## Discussion

This study revealed significant levels of alcohol use, trauma exposure in the workplace and probable PTSD among PSP, and it is one of few studies of this population to measure drug use. It also identified factors associated with substance use, specifically workplace trauma, probable PTSD and gender. These results emphasise the need for interventions designed to ameliorate the effects of workplace trauma exposure among PSP that recognise the interplay of PTSD and alcohol and other drug use (Turner et al., 2018).

The high level of workplace trauma exposure reported by participants is in accordance with Carleton et al.’s (2019) research on Canadian PSP. The most prevalent traumatic events were similar across both studies, specifically: ‘sudden violent death’, ‘sudden accidental death’ and ‘serious transportation accident’. While the current study did not measure the frequency of exposure to each event type, Carleton et al. (2019) reported that PSP were often exposed 11 or more times to the more common events. Given the elevated rate of trauma exposure in this study, it is not surprising that high rates of probable PTSD were also observed, with 39.4% of the sample screening positive. This proportion is much higher than estimates among PSP in Australian (10%; Kyron et al., 2019b) and international research (10%; Berger et al., 2012) and may reflect the PC-PTSD-5’s status as a screening tool with a cutoff that maximises sensitivity (Prins et al., 2016). It also may be the case that as the current study did not randomly sample participants, those who opted-in were more willing to report PTSD symptomology. Nonetheless, it is clear that PTSD is a workplace-related risk for PSP.

This study is one of few to examine prevalence of hazardous and harmful use of alcohol and other drugs among PSP. Consistent with previous research on Australian PSP (Kyron et al., 2019a), rates of problematic alcohol use were higher than those found in the Australian general population, with one-third of participants reporting hazardous and harmful use relative to 22.2% (O’Brien et al., 2020). Alcohol is often relied on as a means to alleviate feelings of distress in PSP (Di Nota et al., 2021) and previous research has highlighted its counterproductive role in exacerbating mental health issues (Turner et al., 2018). Rates of harmful drug use, compared to alcohol use, were lower at 13.3% but still warrant attention in this at-risk population. Although directly comparable data are not available for the Australian general population, this is higher than the national population prevalence of 12 month drug use disorder (3.3%; ABS, 2023).

### Demographic factors associated with harmful alcohol or drug use

No demographic factors were associated with hazardous and harmful alcohol use. The only significant demographic factor related to harmful drug use in the multivariate model was gender, with the odds of harmful drug use among females being five times higher compared to males. This finding contrasts with population surveys, which typically report that men are twice as likely as women to meet criteria for a 12 month substance use disorder (ABS, 2023). This discrepancy may stem from limitations of the DUDIT, which has lower cut-scores to indicate drug-related harm in women, potentially inflating harm (Berman et al., 2005; Dwyer and Fraser, 2017). However, the DUDIT has demonstrated good psychometric properties in a range of samples (Hildebrand, 2015). Although gender was associated with drug-related harm, other factors, including age, rurality, length of service and frontline status were not associated with either alcohol or drug-related harm in the multivariate models. This contrasts with prior research finding higher rates of binge drinking among PSP who are male, older, have longer service tenures and work in frontline roles (Kyron et al., 2019a). However, Kyron and colleagues relied on descriptive statistics and did not use inferential techniques to draw comparisons regarding alcohol and drug use, as used in the present study. In addition, their recruitment strategy differed significantly from this study’s approach, using random sampling techniques and achieving a large sample size (*n* = 14,868). As such, while the current study used advanced statistical methodology, Kyron et al’.s (2019a) findings are likely more representative of the wider population of PSP.

Frontline workers did not report higher alcohol or other drug use compared to non-frontline workers, contrary to expectations that traumatic exposure in the field may lead to substance use to cope. However, the effects of cumulative trauma exposure may emerge later in careers as many senior PSP started as frontline workers and substance use disorders often develop over time. These unexpected findings prompt further research to understand the relationship between demographic and work-related factors and alcohol or drug-related harm among PSP.

### Associations between workplace traumatic events, probable PTSD and harmful alcohol or drug use

A key finding of this study was that experiencing a greater number of workplace trauma types was associated with greater odds of hazardous and harmful alcohol use. This finding emphasises the profound effect that encountering traumatic material while on duty can have on PSP. Past research investigating this association among PSP has yielded mixed results (Andrews et al., 2022; Bartlett et al., 2019; Bonumwezi et al., 2022; Carleton et al., 2019). Workplace trauma exposure was not significantly related to harmful drug use; however, it may have been that employees were reluctant to disclose illegal drug use, or that alcohol is a more culturally acceptable method of stress relief after a difficult shift. More research is needed to further understand the negative effects of workplace trauma exposure and strategies used to mitigate these impacts.

It was found that the odds of hazardous and harmful alcohol use were higher among those with probable PTSD than those without. The same was true for harmful drug use. PTSD symptoms and substance use have been closely linked in PSP populations, including alcohol use among firefighters (Bartlett et al., 2019) and alcohol and drug use among first responders (Bonumwezi et al., 2022). A possible reason for this association is that PSP may engage in substance use as an avoidant coping strategy, which is characteristic of PTSD symptomology (American Psychiatric Association, 2022; Di Nota et al., 2021). These findings underscore the importance of early intervention as an opportunity to mitigate risk before more harmful alcohol or drug use or PTSD develops. Guidelines specifically targeted towards the prevention and treatment of PTSD in PSP suggest systematic screening, for example, with the PTSD Checklist for *DSM*-5 (PCL-5) and recommend addressing cooccurring alcohol or other drug use alongside the treatment of PTSD (Phoenix Australia, 2023). More research should be conducted to ascertain how to best support PSP in the immediate aftermath of a potentially traumatic incident.

### Strengths and limitations

This study has several strengths. First, the demographic and work-based characteristics of the sample closely resembled that of a nationally representative sample of Australian PSP (Kyron et al., 2019a), adding credibility to the external validity of these findings. In addition, this study was conducted by researchers external to the employing public safety organisations, a factor shown to improve the validity of self-reported mental health symptoms by PSP over organisation-led screening (Marshall et al., 2021).

This study also has several limitations. First, the study’s cross-sectional design cannot establish causal relationships between variables. In addition, a convenience sample was utilised introducing the potential for selection bias, wherein those who volunteered may differ from the wider population of PSP. In terms of measures, participants could report the types of traumas they had been exposed to but could not indicate how many times each event had been experienced. Taking frequency of each traumatic event into account, akin to the comprehensive assessment conducted by Carleton et al. (2019), would provide a more accurate picture of trauma exposure. It should be noted that some of the self-report surveys used in this study had specific temporal reference periods (e.g. 4 weeks for the PC-PTSD-5; 12 months for the DUDIT). External factors, such as the timing of the survey during the COVID-19 pandemic or workplace cohort effects of drug use (McEntee et al., 2022) may have influenced findings. Future research should build on this study’s findings, by incorporating random sampling and longitudinal research methodologies to further investigate the role of alcohol and other drug use in this population.

## Conclusion

Overall, this research sheds light on the associations between trauma exposure, PTSD and alcohol and other drug use in Australian PSP. Workers in these roles are critical to maintaining public safety and in protecting their communities; however, they put themselves at risk of mental and physical harm. Addressing mental health and substance use challenges faced by these professionals is essential for ensuring the continued provision of critical services to the community.
